# Controlled Mechanical Motions of Microparticles in Optical Tweezers

**DOI:** 10.3390/mi9050232

**Published:** 2018-05-12

**Authors:** Jing Liu, Zhiyuan Li

**Affiliations:** 1 Institute of Laser and Intelligent Manufacturing Technology, South-Central University for Nationalities, Wuhan 430074, China; jingliu@scuec.edu.cn; 2 School of Physics and Optoelectronics, South China University of Technology, Guangzhou 510640, China

**Keywords:** optical tweezers, mechanical motions, optical force, micromachine, Janus particles

## Abstract

Optical tweezers, formed by a highly focused laser beam, have intriguing applications in biology and physics. Inspired by molecular rotors, numerous optical beams and artificial particles have been proposed to build optical tweezers trapping microparticles, and extensive experiences have been learned towards constructing precise, stable, flexible and controllable micromachines. The mechanism of interaction between particles and localized light fields is quite different for different types of particles, such as metal particles, dielectric particles and Janus particles. In this article, we present a comprehensive overview of the latest development on the fundamental and application of optical trapping. The emphasis is placed on controllable mechanical motions of particles, including rotation, translation and their mutual coupling under the optical forces and torques created by a wide variety of optical tweezers operating on different particles. Finally, we conclude by proposing promising directions for future research.

## 1. Introduction

**Figure 1:**
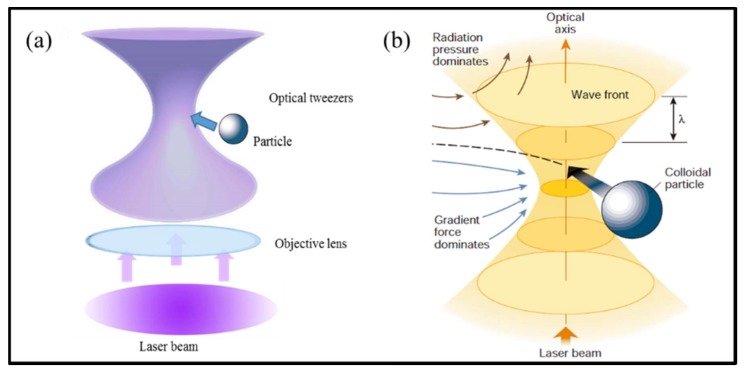
(**a**) A sketch of optical tweezers generated by a strongly focused laser beam to trap objects; and (**b**) a schematic illustration of optical forces exerted on a colloidal particle around the focus spot. (Reprinted with permission from Springer Nature [2].)

Light can drive the mechanical motions of micro- and nano-objects because light carries both energy and momentum that can exchange with these objects. However, this is not an intuitive and obvious concept because the force is extremely weak in usual situations. Thus, in our daily life, optical forces are invisible. Despite this, the light pressure had been hypothesized in the explanation of the nature of comet tails by Kepler (1696). In fact, the electromagnetic theory developed by Maxwell has proposed the optical force, which was described by the term “radiation pressure”. Until the 1960s, the advent of lasers greatly broadened the knowledge on light and made it possible to clearly demonstrate the concept of optical force. In 1986, Ashkin, the pioneer of optical tweezers, creatively used a highly focused laser beam to implement three-dimensional (3D) trapping of dielectric particles around the focus spot [1], as shown in Figure 1a. To understand the principle of this trap (see Figure 1b), the total optical force acting on a particle can be decomposed into two contributions: (1) the radiation pressure, known as the scattering force, which is proportional to the Poynting vector of optical field and points along the direction of the incident beam, tends to destabilize the trap; and (2) the gradient force, which is proportional to the gradient of the light intensity and points toward the tarp focus, confines the particle near the focal spot. The gradient force represents the attraction of particles to the highest intensity region. Thus, the condition for 3D optical trapping is that the gradient force is larger than the sum of the scattering force and stochastic force due to Brownian motion. 

Since then, research on optical tweezers has begun to spread out and expand. Essentially, the basic principle of optical tweezers is the interaction between light and matter, through momentum and energy transfer and exchange, to achieve manipulation of objects. As is well known, optical tweezers can limit objects bound by optical potential well in a small space range. It can trap various objects as small as nanometer-size particles and can exert optical forces with controllable amount at the femtonewton resolution, which is the ideal range for the study of molecule mechanical property. Therefore, optical tweezers have become a promising tool to investigate the mechanical properties of biological specimens, and examples include the force measurements of DNA- and RNA-based motors [3,4,5,6]. This tool has been widely applied to investigate cell cytometry, artificial fertilization of mammalian cells, and even micro-surgery [7,8].

In the early years, the technique of optical tweezers were successfully applied to capture and manipulate micrometer particles [9], even atoms [10]. However, optical forces on the captured objects depend crucially on the interaction between the laser field and the objects themselves. Therefore, multiple degrees and freedoms of control over optical force can be greatly aroused by shaping either the beam or the particles. To achieve various motions of trapped micro-objects, two typical schemes have been successfully adopted in an optical tweezers system. One scheme is to modulate laser beams, for instance using optical vortex beams [11], Bessel beams [12], Laguerre–Gaussian beams (LG beam) [13], and novel beams [14], to build optical tweezers. The other scheme makes use of the special properties of particles, for instance introducing precisely fabricated asymmetric micro-objects [15] rather than usual homogeneous spherical particles, such as micro-turbines or gammadion shaped micro-rotors, birefringent particles [16,17] and Janus particles [18] into an optical tweezers system. These asymmetric particles, when embedded within usual Gaussian or other special optical traps, will exhibit interesting mechanical motion behaviors. Applications have been explored in the fields of Janus motors [19,20], switchable devices [21], and optical probes [22]. Henceforward, optical tweezers have opened research on micro-objects dynamic processes with the aid of high-resolution microscope and laser technologies.

Inspired by the complex organization of biological organisms, the design of artificial micromachines that exhibit controlled mechanical motion and perform sophisticated tasks is an ultimate pursuit of micro-scale engineering. A micromachine allows flexible control of both the direction and speed of motion. At the same time, expanding the scope of their utility in practice has led to the large-scale production of micromachines. Along with the optical tweezers, substantial research on the controlled motion of artificial particles has been undertaken. In the microscopic world, microparticles motions can be strongly modified by external fields and their solution surroundings, such as the temperature distribution and viscosity of the embedded solution (usually water). In addition, due to the random bombardment of the water molecules, the Brownian motions of particles become increasingly significant with the decreasing size of particles. The accurate and comprehensive mathematical description of these forces requires using the rigorous electromagnetic theory to model the interaction between an incident light beam and trapped particles and precisely considering the background medium complex property of particles. However, this can be a daunting task. To calculate the force and torque of trapped particles and their mechanical motion in optical traps, it is crucial to build an appropriate theoretical approach with balance in numerical precision and computational efficiency.

In this article, we discuss how to control the mechanical motions of various particles in optical tweezers under complicated actuation of optical forces and torques by tightly focused laser beams. The rest of this article is organized as follows. In Section 2, we start with description and discussion of the fundamentals of optical forces and different theoretical models and methods, including geometrical optics method, Rayleigh scattering theory and electromagnetic scattering theory used to calculate optical forces. Then, in Section 3 and Section 4, we give a detailed introduction about the development of laser tweezers techniques that have been employed to drive dielectric/metal particles in solution. In Section 5, we focus on a selected topic as optical manipulation of Janus particles, and discuss its potential aspect as micromachines. In Section 6, we continue with a brief summary of optical tweezers in application to the area of biology and physics. Finally, in Section 7, we summarize the state-of-the-art open issues and future directions in optical manipulation of nanostructures.

## 2. Physics and Theory for Optical Forces on Microparticles

Optical forces on particles originate from the interaction of light fields with particles. As the material properties and geometric structures of particles can strongly affect the interaction behaviors, they will also influence both the magnitude and direction of optical forces. To illustrate the basic physics about the optical forces, we take a homogeneous dielectric sphere as an example to discuss how optical forces arise and how they depend on the properties of light–matter interaction. For this special geometry, rigorous analytical solution of light–particle interaction can be obtained from Mie’s theory and its generalized formulation in the framework of classical electromagnetics and electrodynamics. Although the theory and solution are rigorous and reliable, they are quite tedious and troublesome in many situations, and thus some simplification is highly desirable. According to the size of particle in reference to the wavelength of incident laser, solution of optical forces exerted on particle can be categorized into the following three cases: Case (1), the size of particle is far less than laser wavelength; Case (2), the size of particle is much larger than laser wavelength; and Case (3), the size of particle is comparable to laser wavelength. The corresponding methods of optical force calculation are introduced and discussed in details as follows.

(1) Small Particles and Rayleigh Scattering Theory

If the particle is much smaller in size than the laser wavelength (radius less than *λ*/10), one can assume that the external electric field does not vary within the particle. With the decreasing particle size, the wave optical effects gradually come into playing a crucial role. In the optical electromagnetic field with a wavelength far larger than the size of small particles, these particles are often regarded as electric dipoles to account for their optical and mechanical response [23,24,25]. The optical force that acts on a dipole is composed of scattering force and gradient force, which is given by

(1) $$\vec{F}={\vec{F}}_{scat}+{\vec{F}}_{grad}$$

The induced electric dipole will oscillate synchronously with the time-harmonic electric field and emit secondary scattering waves. This process will cause the change of energy flux and amplitude of the incident laser beam, and then results in momentum transfer between light and dipole. Hence, the scattering force acting on small particles can be calculated as,

(2) $${\vec{F}}_{scat}=\frac{n_{1}}{c}C_{scat}\langle \vec{S}\rangle$$

Here, *n*_1_ is the refractive index of surrounding, $\langle \vec{S}\rangle$ is the Poynting vector, and *C_scat_* is the scattering cross section of the particle. The scattering cross section is related with *R* (radii of particle) and *m* (*m* = *n*_2_/*n*_1_) by the following formula,

*C_scat_* = 8/3π*kR*^6^((m^2^ − 1)/(m^2^ + 2))^2^ (3)

where *k* = 2π*n*_1_/*λ* is the wave number of light.

The gradient force exerted on a small particle is proportional to the gradient of the optical field intensity and can be calculated as

(4) $${\vec{F}}_{grad}=\frac{1}{4}\varepsilon_{0}\alpha \nabla {\vec{E}}^{2}$$

where *α* is the polarizability of the particle, and it can be written as *α* = 4π*n*_1_^2^*R*^3^((*m*^2^ − 1)/*(m*^2^ *+* 2))^2^. 

This simple theoretical model can help to understand the basic principle of an optical tweezers. Notice that common optical tweezers are made from a highly focused laser beam (a Gaussian laser beam passing through a high NA microscopic objective and tightly focused) with the focus spot being the center of optical trap. The optical intensity is highest at the focus spot and decays with the distance away this point. Obviously, there is a 3D optical intensity gradient (and thus an optical gradient force) pointing to the focus spot so that a particle will be attracted to the focus spot under this gradient force. Although there also exists a scattering force that tends to push the particle away from the focus spot, this scattering force is much smaller than the gradient force and will not degrade the optical trapping of the particle. Another point is that the gradient force is proportional to the intensity at the focus spot, thus a higher power of laser beam and a smaller focus spot (higher NA of objective lens) would be beneficial for reaching a larger gradient force and stronger optical trapping.

(2) Large Particles and Geometrical Optics Method

For particle diameters larger than 10*λ* (*λ* is the laser wavelength), the geometrical optics method is suitable and widely applied for calculating the optical forces [26,27]. In the geometrical optics regime, the incident light beam is decomposed into several individual rays, each with appropriate intensity, direction, and state of polarization. Each ray can change direction, intensity, and polarization when it reflects and refracts at dielectric interfaces according to the Fresnel formulas. Moreover, reflection and refraction can happen for many times before the intensity of these rays eventually decays to zero. At each reflection and refraction, exchange of momentum between light and particle takes place, and this creates optical force according to Newton’s law of mechanics. The total force on the particle is the sum of all individual forces at each account of interface reflection and refraction for all the rays comprising the incident laser beam. 

**Figure 2:**
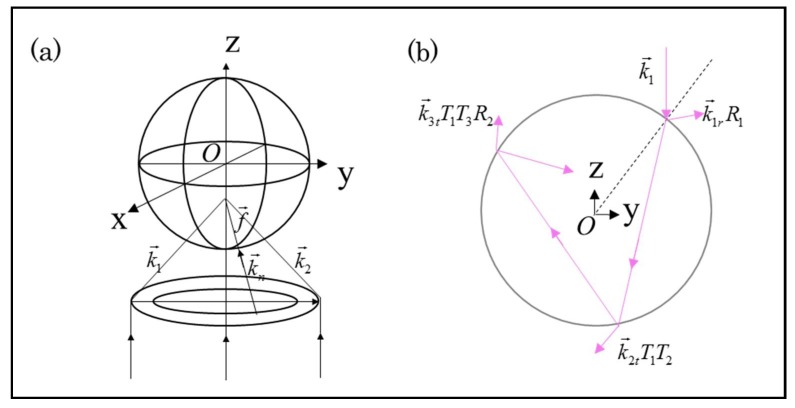
(**a**) The schematic diagram of a Gaussian laser beam tightly focused by a high-NA lens and illuminating the sphere for optical trapping and manipulation. In the ray-optics model, the laser beam is decomposed into a large amount of rays of light denoted by their directional unit vectors ${\vec{k}}_{1}$, ${\vec{k}}_{2}$, …, ${\vec{k}}_{n}$,…. (**b**) Schematic diagram of the ray tracking for a specific ray ${\vec{k}}_{1}$ within the sphere, where a multiple events of ray reflection and refraction take place.

To illustrate this method clearly, the ray-optics model for calculation is shown in Figure 2a. The center of the sphere, point “O”, is set as the coordinate origin. The laser beam is focused by a high NA objective lens to a focal point located at *f* = [*f*_x_*, f*_y_*, f*_z_]. The total light beam is decomposed into a lot of individual rays, and the initial propagation directions of these rays are denoted by ${\vec{k}}_{1}$, ${\vec{k}}_{2}$, ${\vec{k}}_{3}$, …, ${\vec{k}}_{n}$, …, before they interact with the sphere. To describe momentum exchange process in the regime of ray optics, a diagram of the mechanical analysis is illustrated in Figure 2b in detail for clarity. First, consider the force due to a single ray of a power *P* hitting the sphere at the propagation direction ${\vec{k}}_{1}$ with an incident momentum $Pn_{1}/c$ per second, where *n*_1_ is the refractive index of the aqueous solution, and *c* is the speed of light in vacuum. Note that the incident ray will be subject to multiple reflection and refraction events within the sphere, and each time there will be momentum transfer and exchange between light and sphere, generating optical force and torque. Then, the overall force $\vec{F}=\left[F_{x},F_{y},F_{z}\right]$ can be given by $\vec{F}=\vec{Q}\cdot Pn_{1}/c$, where $\vec{Q}=\left[Q_{x},Q_{y},Q_{z}\right]$ is a dimensionless factor that describes the momentum exchange coefficient of each ray interacting with sphere and is associated with the overall reflection of light beam. The factor $\vec{Q}$ is the sum of the reflected ray with directional strength ${\vec{k}}_{1r}R_{1}$ and the infinite number of emergent refracted rays of directional strength ${\vec{k}}_{2t}T_{1}T_{2}$, ${\vec{k}}_{3t}T_{1}R_{2}T_{2}$, …, ${\vec{k}}_{nt}T_{1}T_{n}\left(R_{2}\cdot R_{3}\cdot R_{n-1}\right)$, …. The quantities *T_n_* and *R_n_* represent the Fresnel reflection and transmission coefficients at the *n*th intersection event of the transport ray with the particle surface.

Hence, the overall force contributed by this ray can be calculated via the principle of the exchange of momentum as

(5) $${\vec{F}}_{k_{1}}=\frac{Pn_{1}}{c}\left({\vec{k}}_{1}-{\vec{k}}_{1r}R_{1}-{\vec{k}}_{2t}T_{1}T_{2}-\sum_{n=3}^{\infty }{\vec{k}}_{nt}T_{1}T_{n}\left(R_{2}\cdot R_{3}\ldots R_{n-1}\right)\right)=\frac{Pn_{1}}{c}{\vec{Q}}_{k_{1}}$$

The total force imposed upon the sphere by the focused laser beam is simply the vector sum over the force of all rays of light,

(6) $$\vec{F}=\sum_{i=1}^{N}W_{k_{i}}{\vec{F}}_{k_{i}}=\frac{Pn_{1}}{c}\sum_{i=1}^{N}W_{k_{i}}{\vec{Q}}_{k_{i}}$$

Here, $W_{k_{i}}$ is the weight of contribution parameter of the *k*_i_ ray of light, which is proportional to the intensity profile of the incident laser beam in the entrance pupil of the high-NA lens, and *N* is the total number of rays considered in the calculation.

The above geometrical optics approach clearly illustrates the exchange of energy and momentum between light and particle as the physical origin of optical forces. This method intuitively gives a very simple while quantitative description of optical forces exerted on spheres. More importantly, this method can make theoretical prediction which fits well with experimental data in many studies, for example, optical forces acting on cells [28], deformation of micro-bubbles [29], and Kramers transitions between two optical traps [30].

(3) Mesoscopic particles and electromagnetic scattering theory

For mesoscopic particles with diameters equivalent to or comparable with the laser wavelength, no simple solution of electromagnetic field and optical forces are available, and complicated numerical simulations must be used. Of course, for a spherical paicle, Mie’s theory can be used to yield an analytical solution, albeit still very troublesome. The principle is to calculate the electromagnetic field, construct the Maxwell electromagnetic tensor, use the electromagnetic energy and momentum conservation law, and calculate the optical forces. However, this turns out to be a cumbersome procedure. At present, various numerical algorithms have been developed to handle Maxwell’s equations, such as finite difference time domain method (FDTD) [31], discrete dipole approximation method (DDA) [32], T-matrix method [33] and so on. By using the above methods to obtain the scattering optical field, the optical force exerted on particles can be calculated by

(7) $$\vec{F}=\oint_{S}\vec{T}\left(r,t\right)dS$$

where *S* represents the closed surface surround the particles, and $\vec{T}$ is the Maxwell electromagnetic tensor. Physically, $\vec{T}$ accounts for the connection between optical forces and mechanical momentum exchange [34] through the following formula,

(8) $$\vec{T}=\frac{1}{4\pi }\left[\varepsilon \vec{E}\vec{E}+\vec{H}\vec{H}-\frac{1}{2}\left(\varepsilon {\vec{E}}^{2}+{\vec{H}}^{2}\right)\vec{I}\right]$$

The above briefly discuss the theoretical approaches to understand and calculate the optical forces of various microscopic particles and objects in optical tweezers. This knowledge becomes the physical basis and crucial point for studying the mechanical mechanism of the controlled motion of mesoscopic particles in optical tweezers. Generally speaking, in physics, optical forces not only depend on the optical field but also the particles geometry and physical properties. For simple structures, such as spherical particles and nano-wires, optical forces in practical experiments are basically in accordance with numerical calculation results from the above theories. However, for particles with abnormal geometric shape or material properties, quantitative analysis of the mechanical parameters (position, velocity, force, etc.) has a valuable reference for understanding the experimental observation of various mechanical motions, analyzing the underlying physical mechanism, and constructing the law and methodologies for controlling the complicated motion of trapped particles. On this basis, it is in urgent need to advance and develop optical tweezers theory to a high level that can handle the static and dynamic motion of various particles in diversified optical tweezers with high precision, light computation burden, and deep insights.

## 3. Manipulation of Dielectric Particles 

Light carries energy and momentum. The light–matter interaction is actually a process of energy and momentum exchange. If energy exchange can enormously affect the chemical or physical property of materials, or exert significant optical force and torque, this process will create a new area of engineering science. Nowadays, light is an ideal means to control the motion of objects remotely and wirelessly. Optical tweezers and related optical trapping techniques have been extensively studied and developed as tools for manipulation of particles and for quantitative measurements of forces, torques, and positions in a broad range of multidisciplinary sciences ranging from atomic physics to cell biology [34,35].

On the other hand, considering that the material properties of particles greatly influent optical forces, the trapped particles can be categorized into three types, dielectric particles, metal particles and dielectric-metal particles (namely, Janus particles). In the following sections, we make a brief introduction of the multiple manipulation methods of these particles.

As early as the 1970s, accelerating and trapping microparticles with the force of radiation pressure generated from a continuous laser was achieved [9]. Up to 1986, optical tweezers exploiting a focused laser beam were used to implement 3D trapping of particles [1]. In recent years, inspired by machine miniaturization, there is a widely held and unmet goal to control the diverse motions of nano- and micro-sized particles in a fluidic environment. In the convectional optical tweezers configuration, the optical isotropic microspheres are tightly captured in the symmetrical focused Gaussian laser beam. To break this balance and give these microscopic particles designated complicated motions, the laser beam shaping has been employed to construct various types of optical tweezers and realize fruitful freedoms of mechanical motions of particles.

### 3.1. Novel-Beam Optical Tweezers

**Figure 3:**
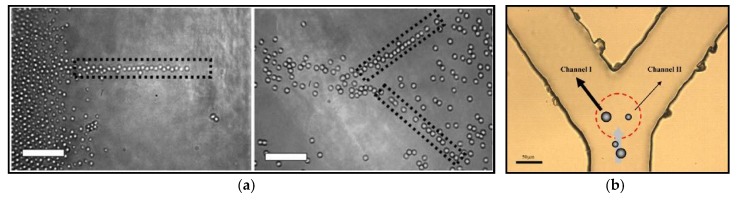
(**a**) Experiments shows herding of polystyrene microspheres using a single optical travellator and two optical travellators. (Reprinted with permission from Springer-Verlag [37].) (**b**) Description of optical sorting different-sizes particles by dual-channel line optical tweezers. (Reprinted with permission form OSA [38].)

Laser beam shaping, the art of controlling the amplitude, phase and polarization profile of a laser’s output, is an extremely useful means to enrich optical tweezers technologies. The first prominent example is illustrated in Figure 3a, where an asymmetric intensity distribution of light field can also generate an asymmetric gradient force [36] to drive particles along a specific direction. Polystyrene microspheres can be trapped and orderly move along the longitudinal optical axis of the line optical tweezers (LOT), like a travellator [37]. Through tilting a cylindrical lens in an off-axis manner, the intensity distribution of LOT is from high to low along the longitudinal direction. Hence, the optical gradient force can propel particles towards the other end of LOT. The second example is illustrated in Figure 3b. Aroused by transporting particles via optical force, the “Y” shape configuration of dual-channel LOTs exhibits the capability of optical transport and sorting different-sizes particles [38]. The magnitude of optical force depends on the particle size and the power of optical field. By adjusting the relative power of two LOTs, optical forces exerted on large-size particles and small-size particles are different from each other. With the assistance of stokes force offered by the stream, the large and small particles will be separated by optical forces.

**Figure 4:**
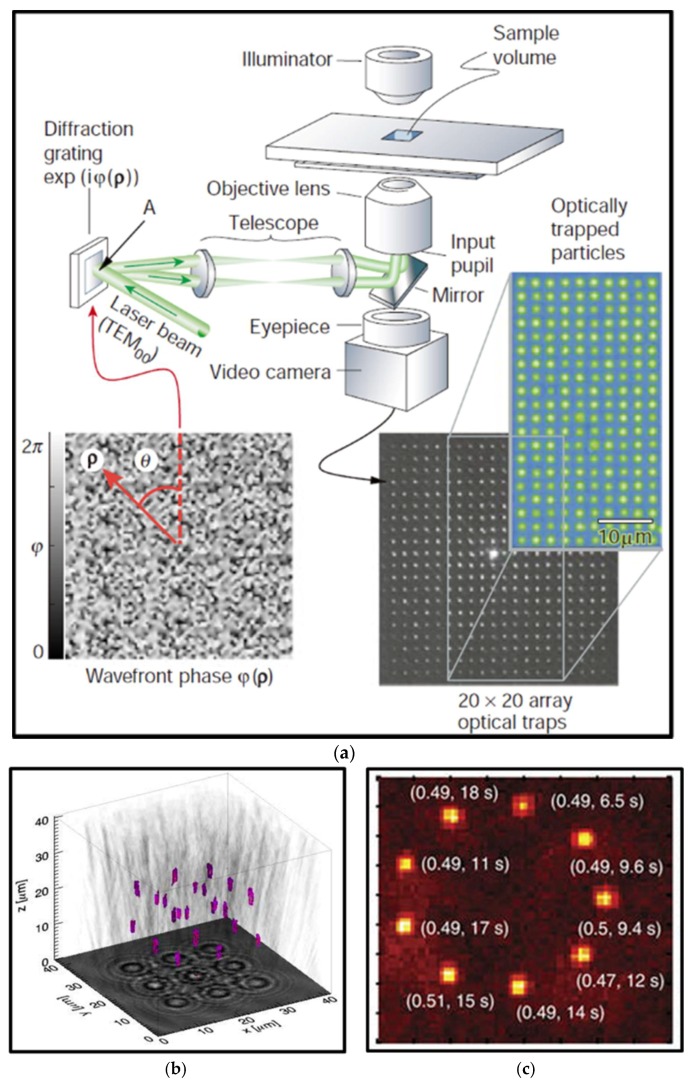
(**a**) Creation of a large number of optical tweezers using computer-generated holograms. (Reprinted with permission from Nature Springer [2].) (**b**) Twenty-two silica spheres are arranged into a crystalline lattice with holographic optical tweezers. (Reprinted with permission form OSA [58].) (**c**) An example of loaded single atoms in a tweezer array. The parenthesis denotes the loading probability and lifetime. (Reprinted with permission from Nature Springer [59].)

Typical controllable methods of particles motions via using optical tweezers are to scan a point-like optical trap with rotating mirrors [39,40,41], galvanometer-driven mirrors [42,43,44], acousto-optical deflectors (AOM) [45,46,47], and spatial light modulator (SLM) [48,49,50]. Due to the optical gradient forces, particles will follow closely with the moving beams. With the advantage of high spatial resolution of positioning, an AOM utilizes the acouso-optic effect to shift the angle of laser beam sent into the objective lens, its focus spot and thus the trap position rapidly, which is well suited for measurements of translation and rotation of biological molecular rotors [47,51]. It is noteworthy that the advent of holographic optical tweezers technology marks the formal entry of 3D multiparticle manipulation into the stage of optical tweezers technology. Various iterative optimization algorithms [52,53,54] have been developed to create specific holography patterns that can modify the amplitude or phase of an input single laser beam to separate equally into a number of laser beams in different directions and form multiple focus spots and optical traps when they pass through the objective lens [2], as shown in Figure 4a. Moreover, it was demonstrated that quasi-statically fast temporal control of computer-generated hologram [55,56,57], together with spatial light modulators, could achieve controlling various motions of multiple particles. In Figure 4b, multiple optical traps can also be applied to organize particles [58] into ordered nanostructures in 3D space, even for atoms [59] as presented in Figure 4c. This technology promotes applications in 3D cellular constructs [60], assembly of structures [61] and individual particle control [62,63,64].

The optical field in usual optical tweezers is basically a low-order fundamental mode Gaussian beam, which is easy to perform but greatly limited in the range and depth of particle capture. With the advent of various laser beams, such as the optical vortex beams, Laguerre–Gaussian (LG) beams, Bessel beams, self-bending beams, and self-accelerating beams [49,65,66,67], plenty of methods to achieve complex manipulations have been proposed. In particular, “non-diffracting” Bessel beams have been exploited to trap atoms and microscopic particles in multiple planes, and construct conveyor belts for them [68]. Because of its non-diffracting effect, the center spot size and shape can remain unchanged in the propagation process. Hence, Bessel beams are widely used to guide the particles along the direction of transmission.

**Figure 5:**
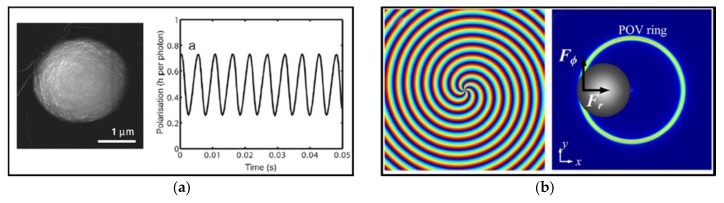
(**a**) Rotation of a vaterite crystal through the transfer of optical spin angular momentum of circularly polarized light. (Reproduced with permission from APS [73].) The left panel shows the scanning electron microscope (SEM) of a vaterite crystal, and the right panel shows the signal recorded by the linear polarization measurement apparatus during rotation of the vaterite crystal. (**b**) Rotation of low-refractive-index hollow glass spheres via the transfer of optical orbital angular momentum of an optical vortex. The left panel represents generation of quasi-perfect optical vertex, and the right panel shows the simulated focal-plane intensity distribution and the forces experienced by the microparticle. (Reproduced with permission from OSA [74].)

In addition to relying on the linear momentum exchange of light with particles for 3D manipulation of particles, researchers also use the angular momentum of light as a new freedom to capture and even drive particles in different ways. The angular momentum of light has two forms, spin angular momentum (SAM) and orbital angular momentum (OAM). Both SAM and OAM of light can be conveyed to particles through absorption and scattering, and result in torque that rotates the objects in addition to the usual trapping operation [69,70,71,72]. As shown in Figure 5a, the transfer of SAM from circularly polarized light to nearly perfectly spherical vaterite crystals, which has similar birefringence properties to calcite [16], can cause this crystal to rotate in a speed up to 1000 Hz [73]. Figure 5b describes that a quasi-perfect optical vortex beam, generated by a spatial light modulator, transfers OAM of light to support the rotation of particles [74].

**Figure 6:**
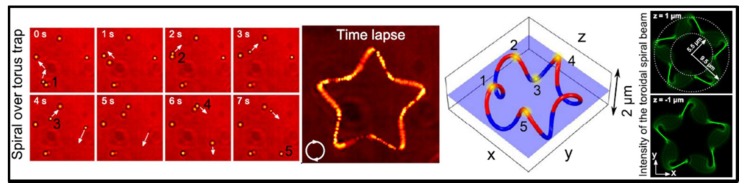
The optically induced particle motion in a 3D toroidal-spiral laser trap. The time-lapse image shows the particle flow revealing a starfish shape, which is corresponding to the shape of the trapping beam in right panel. Top and bottom corners of the 3D toroidal-spiral curve are plotted in red and blue, respectively. (Reproduced with permission from OSA [77].)

A significant progress is the discovery that both intensity gradient and phase gradient of light can be utilized to drive objects. To deal with various future specified tasks, the objects should be controlled to follow well-designed complicated trajectory of mechanical motion. Therefore, both intensity and phase gradient forces are crucial for the construction of complicated 3D optical traps. Specifically, the focused Gaussian vortex beam is of this kind. The intensity gradient force of this beam allows for 3D trapping, while the phase gradient force created by the vortex beam propels the particle to rotate around the ring [75]. With the development of holographic technique for 3D beam shaping, a lot of freestyle optical traps that satisfy the above mentioned gradients can stably trap particles and propel them to move [76]. Figure 6 displays the optically induced particle motion in a 3D toroidal-spiral laser trap, with the distance up to 25 μm deep from the highest to the lowest. Note that these laser traps have various types, including, but not limited to ring, triangle, square and 3D curves [77].

### 3.2. Near-Field Optical Tweezers

As conventional optical tweezers work based on the far-field technique with optical lens or microscope objectives used to focus an incoming laser beam into a tiny spot, the spatial confinement of these far-field optical tweezers is inevitably limited by the diffraction effect of light, namely, the Rayleigh diffraction limit of microscope resolution. In addition, the gradient force is proportional to the third power of the particle radius, and this intrinsically limits the ability to capture and position nanoscale objects because in these situations the optical trapping force is not sufficiently large to overcome the escape force imposed by the random Brownian motion of water environment. It is worthwhile to mention that optical manipulation of metal nanoparticles have been achieved due to its high absorption effect [78,79,80]. However, the characteristics of most biomolecules, such as proteins and carbohydrates, are closer to dielectric particles. As a result, it is not feasible to use conventional microscope optical tweezers to trap these macromolecules. Alternative route must be explored to solve this problem. 

**Figure 7:**
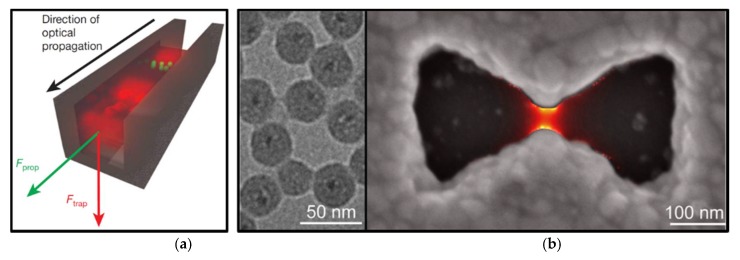
(**a**) Schematic of the slot waveguide used to transport small particles via evanescent field. (Reproduced with permission from Springer Nature [85].) (**b**) Schematic of the bowtie apertures used to trap small particles via plasmons. The left panel represents the transmission electron microscope (TEM) image of the silica-coated quantum dots used in trapping. (Reprinted with permission from [87]. Copyright 2016 American Chemical Society.)

In 1997, Lukas Novotny proposed a method to irradiate the metal tip with laser and achieve significant enhancement of evanescent field [81]. Unlike propagating fields used in conventional optical tweezers, the energy of evanescent field is spatially concentrated in the vicinity of the light source and extends from the interface up to several hundred nanometers away in distance. As the intensity distribution of light decays rapidly with a length far smaller than half the wavelength (the scale of conventional optical trap) this generates a very strong gradient force enabling to capture nano-scale particles [82,83,84]. As shown in Figure 7a, a well-designed subwavelength waveguide slot illuminated by laser beam can excite a strongly localized field at its center. This localized field can capture 75 nm dielectric particles and *λ*-DNA, and simultaneously utilize the scattering and absorption forces to transport particles along the light propagation direction [85]. However, such evanescent fields are difficult to capture metal particles, as this structure strongly scatters and absorbs light.

With the development of nanofabrication technology, plasmon nano-tweezers, which depends on the surface plasmon polaritons (SPPs) of a metal-dielectric interface, is particularly efficient in confining and localizing light down to the nanometer scale [86]. Plasmonic nanostructures can be engineered to couple with propagating light and concentrate it into tightly localized optical fields. Figure 7b shows bowtie apertures designed and fabricated to trap 30 nm insulated QDs, yielding a system with stable single particle trapping [87]. To enhance the interaction between the microscopic particle and electromagnetic waves, it was demonstrated that the SPP of wrapping dielectric particles with black phosphorene (BP) layers excited by the LG beam can be applied to achieve tunable optical force [88]. The combination of plasmon optics and optical manipulation has opened up new opportunities for optical trapping at the nanometer scale.

## 4. Manipulation of Metal Particles

Dielectric particles such as polystyrene or silica particles are often used as carriers or handles to measure the mechanical properties of bio-molecules. By contrast, trapping metal particles still faces intractable problems, due to strong scattering force and severe optical heating effect [89], especially when the laser wavelength is close to the surface plasmon resonance (SPR) of the particle. When the size of metal particle is close to or larger than the wavelength of light, scattering force increases with particle size much faster than the gradient force. Therefore, these mesoscopic metal particles trend to escape from the optical trap, and only the small metal particles with sizes well below the Rayleigh diffraction limit can be stably trapped.

Among metal particles, due to wonderful stability and excellent compatibility to biological molecules, gold nanoparticles are widely used in the areas of data storage, bio-sensor, and surface Raman scattering enhancement. For example, gold particles as local heat sources can cause hybridization of DNA [90], as “plugs” connecting enzymes to electrical nano-circuits [91]. For optical tweezers system, it is crucial to trap particles more easily and quantify optical forces exerted on trapped particles. Thanks to the advancement in optical engineering, methods to generate optical fields with inhomogeneous spatial distribution have been developed [71,83,92,93,94,95]. For example, Bessel beams [96,97] enable trapping metal nanoparticles [98], even under the SPR condition, by tailoring the spatial distribution of the vectorial optical field [99].

**Figure 8:**
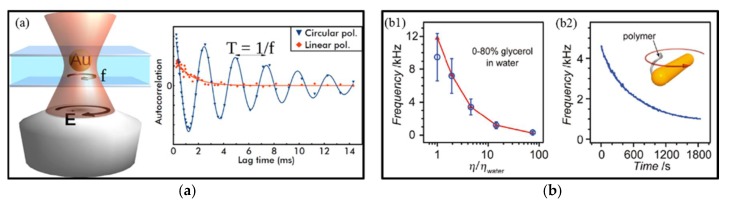
(**a**) Ultrafast spinning of gold nano-particles in circularly polarized optical tweezers, and the right panel displays the intensity autocorrelation functions for a particle subject to circularly (blue squares) and linearly polarized laser light (red triangles). The rotation frequency *f* can be obtained by fitting the experimental data. (Reprinted with permission from [70]. Copyright 2013 American Chemical Society.) (**b**) Gold nanorod motors for localized environment sensing: (**b1**) the rotation frequency of god nanorod motors versus solution viscosity relative to water; and (**b2**) the rotation frequency of the gold nanorod with attached molecules decreases when time goes by. (Reprinted with permission from [100].)

In addition, it was demonstrated that gold nanoparticles could absorb spin angular momentum from circularly polarized light to rotate at high frequencies of several kHz [70] (see Figure 8). For gold nanorods, the speed could even reach a very high rotation frequency up to 42 kHz in water [79], much faster than previously reported results of optical spinning. This ultrafast rotation of particles is highly dependent on the surrounding environment, so it could be useful for probing localized viscosity and temperature [100], as shown in Figure 8. In comparison to trapping in water, the manipulation of metallic nanoparticles in air has been reported [101]. However, this faces more challenges due to faster Brownian motion of aerosols and higher heat dissipation. Thus, it will be quite interesting to further explore many exciting fields such as heat transfer at solid–gas interface at the nanoscale and irradiation of metallic particles in air or vacuum.

**Figure 9:**
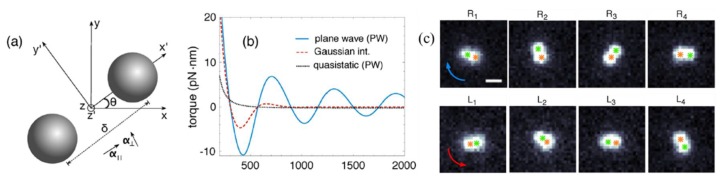
Rotation of an electrodynamically bound nanoparticle dimers (EBD). (**a**) Schematic and coordinate system of an EBD. (**b**) Calculated torque on the EBD as a function of the center to center separation of the two 150 nm diameter Ag nanoparticles. The solid blue curve shows the torque calculated by assuming that the incident source is a plane wave, the dashed red curve is for a source with a Gaussian intensity envelope, and the dotted black curve is the torque calculated under the quasi-static approximation for an incident plane wave. (**c**) Series of experimental dark-field images (ordered from top to bottom in time) of an optical dimer composed of two 150 nm diameter Ag nanoparticles rotating clockwise and counterclockwise for right-handed and left-handed circularly polarized light, respectively. Arrows designate the direction of rotation of the EBD. (Reprinted with permission from [102]. Copyright 2017 American Chemical Society.)

Besides investigating the simpler case of spherical metal particles, metal nanorods and metal nanowires, many recent papers have begun to consider metal particles with complex structures in an optical trap. Shaping gold nanoparticles into nano-prisms can result in an increase in the trap stiffness by an order of magnitude as the destabilizing scattering force is reduced. Optical tweezers also support the study of the dynamics of two or multiple interacting particles. In Figure 9, two 150 nm in diameter Ag nanoparticles with small separations are combined into an electrodynamically bound nanoparticle dimer structure (EBD structure) [102]. The EBD rotates clockwise and counterclockwise under the right-handed and left-handed circularly polarized light, respectively. Through dynamics simulations and experiments, the results demonstrate that negative torque could create new opportunities to control the orientation of dimer structures by interparticle separation. 

As metal nanoparticles support SPR [103,104], this characteristic has profound influence on their interaction with light, including strongly enhance light absorption and light scattering. For example, plasmon excitation induces strongly amplified optical near-fields near the metal surface, so that the trapped nanoparticles can be used as antennas to effectively couple light with molecules or other objects. Moreover, the SPR properties of metal nanoparticles, including the resonance wavelength, charge displacement feature, near-field enhancement, and plasmon damping characteristics, can be precisely tuned via particle properties design, in particular, by selecting the particle shape, size and composition.

**Figure 10:**
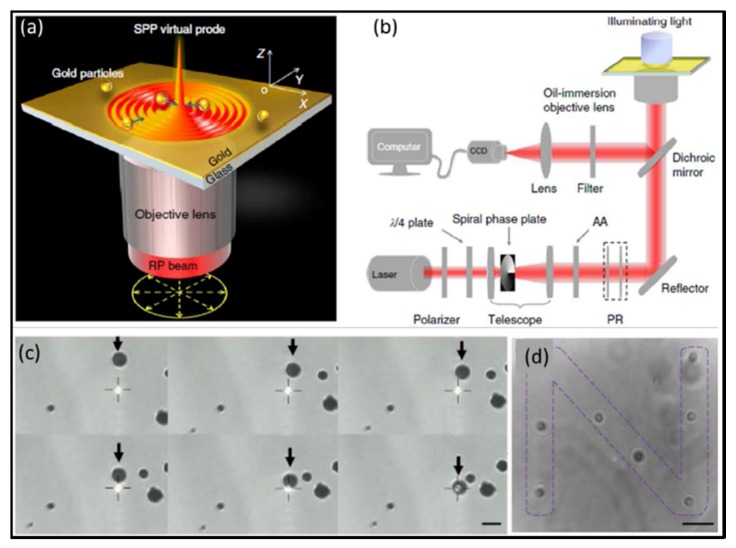
Experimentally setup and trapping results of microscale metallic particles: (**a**) schematic of trapping metallic particles by a SPP virtual probe; (**b**) experimental set-up of the focused plasmonic trapping system; (**c**) successive images of gold particles (diameter of 2.2 ± 0.1 μm) trapped by the focused plasmonic tweezers with a time interval of 0.5 s; and (**d**) patterns for the letter “N” constructed by gold particles (diameter of 1 ± 0.1 μm) in the focused plasmonic tweezers. (Reprinted with permission from Springer Nature [105].)

Plasmonic tweezers, which work based on SPPs excited in metallic nanostructures, have exhibited an enhanced attractive force for particles [31]. SPPs are the collective oscillatory behaviors of free electrons that occur when the electromagnetic waves propagate on a metal-dielectric interface so that the free electrons and incident photons of the metal surface are strongly coupled with each other. This kind of behavior can limit the light field to the deep subwavelength scale and thus break down the optical diffraction limit. The local evanescent field intensity induced by the SPPs is greatly enhanced compared with the incident light. Moreover, intensity enhancement factor can reach 10^3^ orders of magnitude. In 2013, Yuan et al. [105] utilized the SPPs excited on the gold film by an incident objective lens focused radially polarized beam to implement the manipulation of mesoscopic metal spheres (about 0.55–2 μm in diameter), as shown in Figure 10.

## 5. Manipulation of Janus particles

The above sections have clearly shown that both dielectric microsphere and metal nanospheres can be firmly captured in the conventional optical tweezers and become rest eventually right at the focus spot or more precisely the minimum position of optical potential despite of their initial position and velocity. Thus, it is difficult to observe self-propelled mechanical motions of nano- and microparticles in microscopic world via optical tweezers. However, a special kind of particles, called Janus particles involve both dielectric and metal materials, naturally provide a route to break down the structure symmetry. In this section, we will give a detailed introduction about the diversified and fruitful mechanical motions of dielectric-metal Janus particles in various optical traps.

### 5.1. Mechanical Motions of Janus Particles in Optical Fields

**Figure 11:**
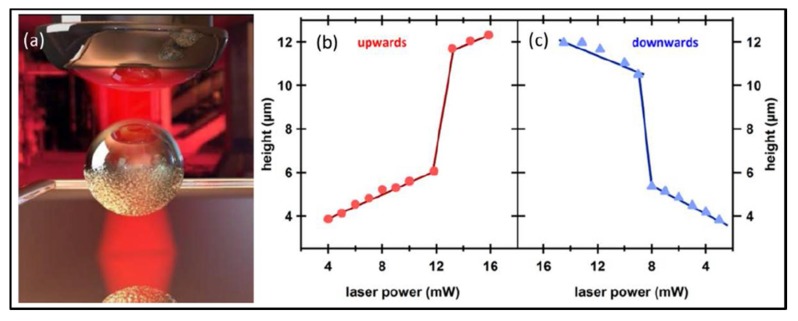
An upward/downward jump of Au/SiO2 Janus particle in a weak focused optical trap with gradually increasing/decreasing laser power. (**a**) Schematic of the Janus particle in a trap. Axial displacement as a function of (**b**) increasing and (**c**) decreasing trapping laser power. (Reprinted with permission from [106]. Copyright 2015 American Chemical Society.)

As Janus particles involve multi-functional materials simultaneously within an individual micro-object, the interactions between Janus particles and optical fields are complicated, and even can bring incredible novel effects. In 2015, Spas Nedev et al. observed an upward/downward jump of Au/SiO_2_ Janus particle (1.3 μm-diameter SiO_2_ sphere half-coated with a 5 nm Au film) in a weak focused optical trap with gradually increasing/decreasing laser power [106], as presented in Figure 11. Given that the gold film is up to 5 nm in thickness, it exhibits not only optical effect but also thermal effect. When the laser power increases, optical gradient force immediately becomes larger, and the thermophoretic force does not respond immediately due to the delayed thermal accumulation and temperature increase response, so during this period the Janus particles are immediately pulled towards a higher stable position by the optical gradient force. During this process, the thermal accumulation continues and temperature still increases to a certain value. When the laser power decreases, optical gradient force decreases, but thermophoretic force still maintains as it is. As a result, the particles are pushed to a lower position. Afterwards, this team used Janus particle as a handle and achieved complete extension of the DNA tether by tuning the laser power instead of moving particles [107].

**Figure 12:**
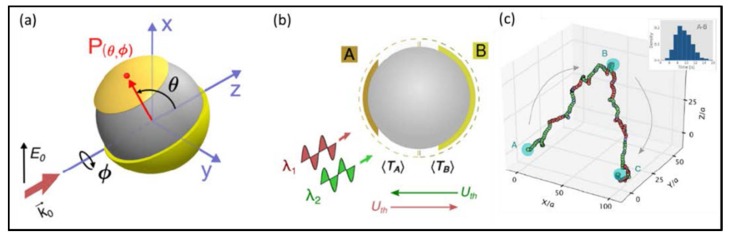
Transport a gold/titanium-nitride Janus particle by switching the two wavelength of light. (**a**) 3D view of an asymmetric particle consisting of a uniform dielectric core and two opposing caps. (**b**) The cross-section of a polystyrene particle with a titanium-nitride (A) and gold (B) cap. (**c**) Example of a composite asymmetric particle transported along the target A-B-C route, by switching the wavelength of the actuating light: 800 nm (red circles) and 500 nm (green circles). (Reprinted with permission from [108]. Copyright 2016 American Chemical Society.)

More intuitively, Ilic devised a hamburger-type Janus particle, as shown in Figure 12. The particles use a dielectric material sphere as the substrate, one end coated with a gold film and the other end coated with a titanium nitride film. Since these two faces (gold and titanium-nitride) are designed to preferentially absorb light of different wavelength, regardless of the particles orientation, it allows for bidirectional motion. By simply turn on and off these two beam separately, one could achieve to drive a gold/titanium-nitride Janus particle to any position in 3D space [108].

### 5.2. Rotation and Translation in the Point and Line Optical Tweezers

**Figure 13:**
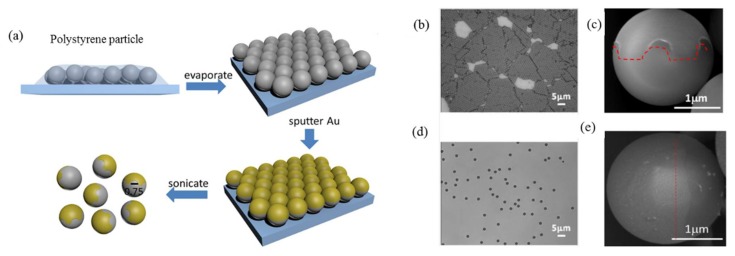
(**a**) Fabrication of Janus particle via magnetron sputtering methods; (**b**,**d**) the micrographs of dried polystyrene particles at high and low concentrations on a glass substrate; and (**c**,**e**) SEM images of Janus particles with patterned dividing line and flat dividing line. (Reprinted with permission from [110]. Copyright 2015 American Chemical Society.)

**Figure 14:**
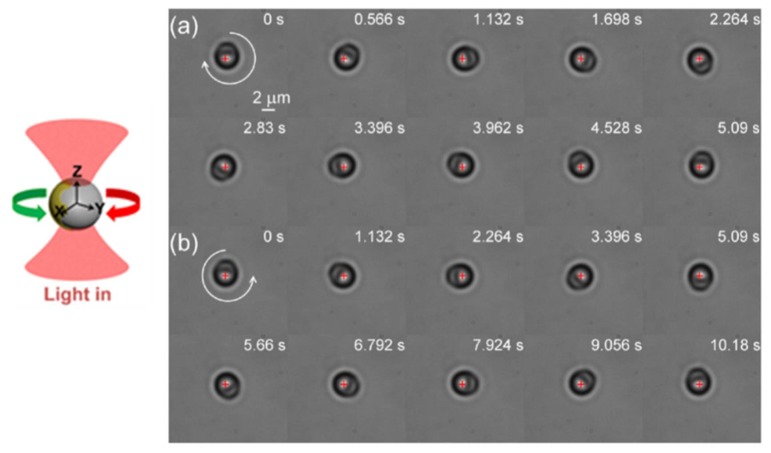
The bi-stable rotation of patterned Janus particles in point optical tweezers: (**a**) the clockwise rotation, laser power *I* = 57 mW, objective NA = 0.7; and (**b**) the counterclockwise rotation, laser power *I* = 28.5 mW, objective NA = 0.7. (Reprinted with permission from [110]. Copyright 2015 American Chemical Society.)

Different to pure metal or pure dielectric particles, Janus particles constitute a rich model system for investigating the optical and mechanical interactions between matter and light. More interestingly, David G. Grier’s group found that rather than wandering randomly, a Janus particle circulates back and forth through a diverging laser beam [109]. In 2015, we achieved the controlled stable rotation of a Janus particle in linearly polarized point optical tweezers by introducing patterned metal coating [98,110]. The fabrication of Au-PS Janus particles by half coating polystyrene spheres (several micrometers in diameter) with a gold thin film (several nanometers in thickness) via magnetron sputtering technique, is illustrated in Figure 13a. In experiments, we had obtained two kinds of Janus particles by controlling the concentrations of polystyrene spheres. They are Janus particle with patterned dividing line and with flat dividing line, as shown in Figure 13b–d. When illuminated by a focused laser, the patterned Janus particles in water can stably rotate around the optical axis. A series of snapshots of clockwise and counterclockwise rotation of a Janus particle are displayed in Figure 14. The brighter part of the sphere corresponds to the uncoated hemisphere of the polystyrene. The Au-coated hemisphere appears darker in the images, as it transmits less light. Both the rate and the direction of Janus particles can be flexibly controlled by adjusting the position or intensity of the focused laser beam. On the other hand, when capture a Janus particle with flat dividing lines, does not show any sustained directional rotation, only randomly vibrates near the trap center.

**Figure 15:**
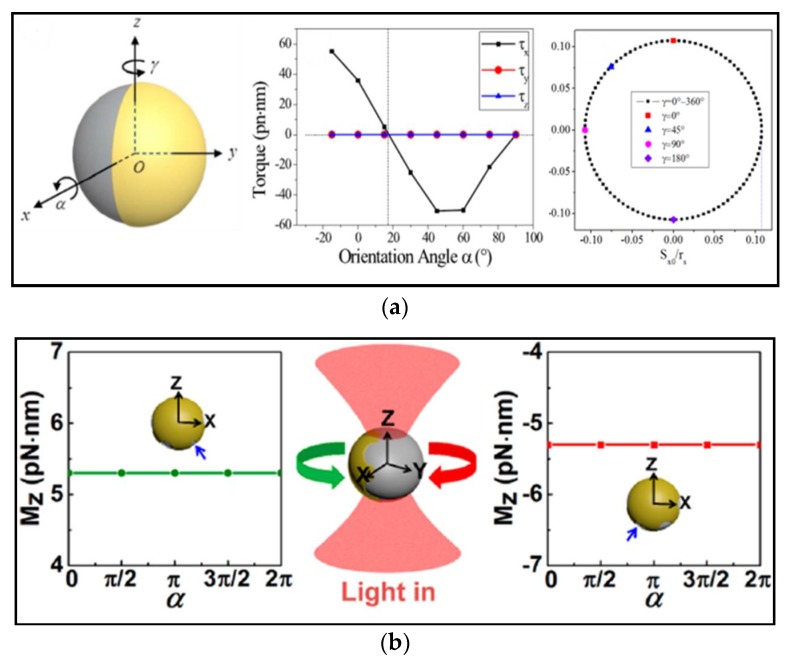
(**a**) Ray-optics model for optical force and torque on a spherical metal-coated Janus particle. (Reprinted with permission from OSA [27]). (**b**) The schematic shows that Janus rotor with patterned metal coatings can be controlled to rotate in optical tweezers. (Reprinted with permission from [110]. Copyright 2015 American Chemical Society.)

To better understand the underlying physics and for deeper insight toward exploring novel ways to manipulate the mechanical motion of microscopic objects, theoretical and numerical analyses are necessary. Considering the huge computation caused by the complicated structure of Janus particles, we proposed a theoretical method based on a ray-optics model to calculate the optical force and torque in a Janus particles in optical tweezers [27], as illustrated in Figure 15a. Numerical analyses show that spontaneous symmetry breaking induced by the pattern of metal coatings on particles plays a critical role to bi-stable rotation of Janus particles, as shown in Figure 15b. Thus, instead of relying on precise fabrication of device, the inevitable fabrication of Janus particles brings potential prospects in producing the millions of controllable microdevices.

**Figure 16:**
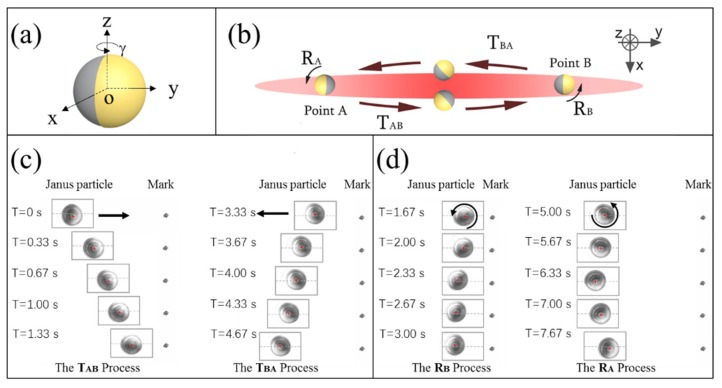
The self-propelled cyclic round-trip motion of a metallo-dielectric Janus particle in static line optical tweezers. (**a**) A 3D stereogram of the Janus particle made from a PS bead half-coated with thin gold film. The Au–PS separation plane of the particle can rotate around the axis of the laser beam (*z*-axis). (**b**) A schematic illustration of the roundtrip motion of a Janus particle in LOT. (**c**) The experimental translational process of a Janus particle moving from Point A to Point B, and from Point B to Point A. (**d**) The experimental rotation process pf a Janus particle rotating around the light propagation direction at Point A and at Point B. (Reproduced from [18] with permission from The Royal Society of Chemistry.)

Nowadays, it is highly desirable to entitle these microscopic particles with designated rectilinear and rotational motions as complicated and general as possible by using as simple as possible static optical field. However, the mentioned rotations of Janus particles are relatively simple and intuitive. Full potential of strong coupling in rotational and translational motion is the core value of Janus particles. Figure 16 displays the self-propelled cyclic roundtrip motion of a metallo-dielectric Janus particle in a static line optical tweezers (LOT) [18]. This complicate mechanical motion can be decomposed into the translation process and the rotation process. In translation process, the Janus particle moved along the straight line, and its Au-coated surface was on the opposite side to the movement, just like a propeller. In rotation process, when it arrived at the target point, it rotated about a semicircle around the optical axis slowly but automatically, just like a rotator. The key to achieve this motion lies in the collective and fine action of the linear and angular momentum exchange between the particle and the LOT field. At this time, the roles of Janus particles are not only the moving particles, but also self-adaptive-optics microdevices. As no external perturbation and manipulation is inputted, the whole self-adaptation is smart enough to allow for formation of such a complicated cyclic round-trip motion with non-contact technique in microscopic world.

With the development of micro- and nanofabrication technology, the compound Janus particles promote magnificent prospects in biomedical fields. Zhao et al. investigated that PH-responsive polymeric synthesized Janus containers for controlled drug delivery process [111]. The Janus containers can selectively load oil-soluble materials into their hydrophobic cavities and release them by adjusting the PH environment. Zhang Jian et al. used semi-wrapped gold film Janus particles as a reflection contrast agent injected into optically transparent zebrafish, and found that the OCT signal of zebrafish was significantly enhanced [62]. Similar to spherical mirrors, Janus particles have the ability to reflect light, which can more effectively participate in the optical imaging process than pure metal particles or dielectric particles [112].

## 6. Applications

**Figure 17:**
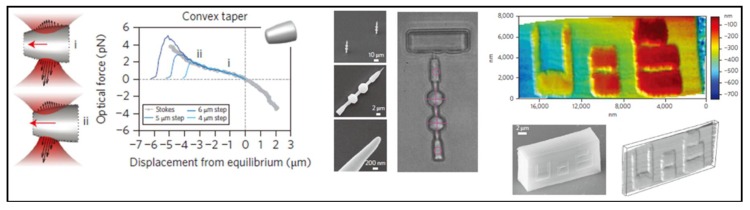
(**Left**) Experimentally measured force-displacement profiles for a particle with a convex taper; (**Middle**) SEM image and optical image of probes, respectively; and (**Right**) a shaped particle used as a scanning probe to image surface topography. (Reprinted with permission from Springer Nature [115].)

Optical tweezers have become a powerful tool for manipulating nanometer-sized and micrometer-sized objects including biological cells and particles [35,113]. Recently, with the aid of modern position analysis apparatuses, optical tweezer technology has proven to be an ideal tool to trap a variety of objects wirelessly and control them to serve as a highly sensitive force transducer. An optically trapped sphere is an elegant example of a microscopic harmonic oscillator, which is capable of measuring femtonewton-scale forces [114]. Besides capturing particles as a handle for molecule force measurement, optical tweezers can assist accurately to locate or detect surface morphology. For example, fabrication of needle-like particles has been illustrated as a scanning probe to image nanoscale structure surface topography wirelessly with the lateral resolution of 200 nm and the depth resolution of ~10 nm, as can be seen in Figure 17 [115]. Mechanical analysis of this structure shows that optical force is linear with its trapped position. The properties of optical force density show the potential to obtain qualitative insights into the behavior of shaped dielectric particles in optical fields [116]. Inspired by complex organization of biological machines, researchers have developed similar artificial micromotors, driven by light field, to achieve self-driven motions. Because of the small size and strong loading capacity, micromotors have emerged and advanced quickly in the fields of drug delivery [117], biomedicine [118] and chemical analysis [119]. However, the optical torque is not as easy to control as optical force exerted on particles. In early days, it was found that optical torques could exert on a particle whose symmetry was lowered either by shape modification [120,121,122,123,124] or by refractive index anisotropy [16,51]. Due to high accuracy and simple operation of 3D laser direct writing technology, fabricating complex microstructures has become popular. A series of well-designed micromachines have been proposed in recent years. The common light-driven structure is Archimedes screw, which has been used for micropumps [125]. However, due to the light diffraction limit, a range of primary interest for nanotechnology have not been widely exploited.

**Figure 18:**
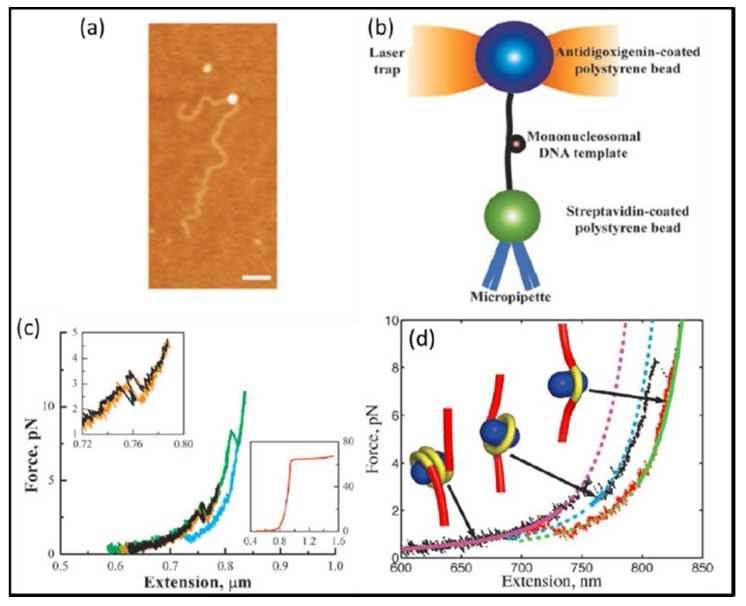
(**a**) The antidigoxigenin-coated polystyrene bead was held in the optical trap, whereas the streptavidin-coated polystyrene bead was held onto a micropipette via suction. (**b**) Schematic of stretching nucleosome-armed DNA strands with optical tweezers. (**c**) Representative force-extension curves of the mononucleosome. Black and green indicate when the fiber was being pulled; orange and blue indicate when the fiber was being relaxed. (**d**) Probability of the first wrap unraveling as a function of force was obtained by summing a normalized histogram of the first wrap opened vs. force. (Reprinted with permission from [140], Copyright 2006 National Academy of Sciences.)

Accurate measurement and determination of various critical mechanical quantities, such as position, velocity, force, power, energy of microscopic biological systems in cells, such as DNA, RNA, enzymes, proteins and other macromolecules, larger cell organelles such as mitochondria, chloroplast, chromosome, and so on, and macroscopic cell infrastructures such as nucleus, cytomembrane, and others, are extremely useful and important to understand the connections among energy, information, and life in cells. Fortunately, optical tweezers have advantageous features including position sensitive detector with nanometer accuracy, high-precision force transducer (ranging from 0.1 pN to 100 pN), and compatibility with liquid medium environments, which make it highly suitable for application in biological studies. In the present, optical tweezers has been assumed as an important tool for studying the kinetics of single molecules and motor proteins [126]. Enormous progress has been made, for instance, it was found that a force of 1000 pN is sufficient to break down the covalent bond and enough to separate two mammalian cells; a force of 30 pN will stop DNA helicase and polymerase form working and is enough to overcome the thrust of bacterial flagella; a force of 10 pN can stop myosin and drive movement of proteins and dynein [127]. One of the most remarkable achievements is that Wang et al. used optical tweezers to measure the elastic modulus of DNA with high accuracy [128]. On the other hand, many scientists have used similar techniques to study RNAP, siRNA, DNA polymerase [129,130,131,132], microtubule, toroidal T7 helicase, the ribosome, nucleosomes [133,134,135,136], overstretching B-DNA, reversible unfolding RNA and bacteriophage ϕ29 [137,138,139]. As illustrated in Figure 18, Mihardja et al. [140] studied the interaction between nucleosome-armed DNA strands and histones with optical tweezers. It is a pity that all the mechanical processes of measurements are in the non-physiological state. Nowadays, the measurements of biomolecular mechanical properties under physiological conditions have been recognized as a magnificent goal in the academic research.

In addition to accurately measuring the mechanical properties of biological macromolecules, optical tweezers have earned their spurs in manipulating organelles in cells. In the past, biological application with optical tweezers was limited to single molecule and single cell research in vitro. However, the life performance of living beings always hides in the environment between cells and solvent. For example, how cells generate, migrate, or interact between cells and proteins in living animals remains to be a critical problem in life science. Therefore, it is in urgent need to explore and figure out how various biological cells work systematically together. Based on the current microscope technology, with the extremely complicated system in vivo, it is very difficult to manipulate living cells. Thus far, the research of biological cell technology in vivo is one of hot spots in optical tweezers.

**Figure 19:**
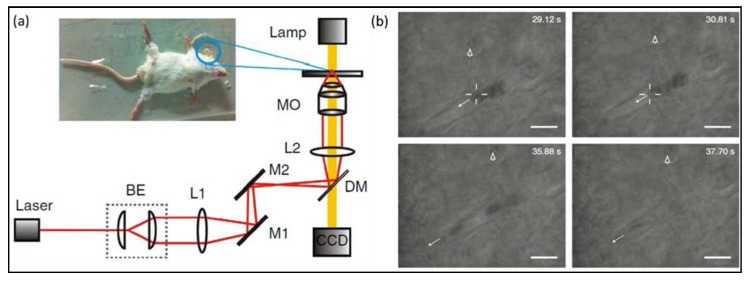
Optical trapping of red blood cells within living mice. (**a**) The optical tweezers setup. The inset shows the ear of the mouse at the sample stage. (**b**) Optical tweezers trap the red blood cells, which have blocked the capillary, and remove them from the capillary. (Reprinted with permission from Springer Nature [141].)

In 2013, Li’s research group demonstrated that they achieved optical capture of cells in living animals [141] with optical tweezers technology, as displayed in Figure 19. Red blood cells in vivo were captured and manipulated for the first time. In this experiment, the laser penetrates the dermis of the mouse ear and reach the capillaries with a depth of 50 microns. In addition, they utilize the optical traps to gather red blood cells and artificially create clogged blood vessels. Inversely, they used optical tweezers to manipulate aggregated cell clusters in blood vessels by dragging one of cells to restore normal blood flow. This achievement for the first time implements non-contact vascular dredging operation in vivo. This work pioneers to study a new field of living animals with optical tweezers and provides a completely new technical method for living research and clinical diagnosis. Subsequently, in 2016, Koster et al. achieved manipulating nanoparticles and cells in live zebrafish via optical tweezers, analyzed the adhesion properties of endothelial cells and macrophages, and probed the characteristic of membrane deformation [142]. These results show great potential of optical tweezers in studying life science in vivo.

## 7. Conclusions and Perspectives

As a rapidly developing research field, optical tweezers create exciting possibilities for nanometer-position-sensitive detection, piconewton-scale force measurements, and even self-propelled micromachines. In this paper, we briefly introduce the development and principle of optical tweezers. Essentially, optical tweezers are a manifestation of optical force that originates from the energy and momentum exchange between light and particles. Based on the size of particle in reference to the wavelength of laser beam, the theoretical methods of the optical force exerted on trapped particles in optical tweezers have been categorized into the following three cases: (1) small particle and Rayleigh scattering theory; (2) large particles and geometrical optics method; and (3) mesoscopic particles and electromagnetic scattering theory. Then, we show that the diversified developments of optical tweezers, such as novel optical beam optical tweezers and near-field optical tweezers have been made in addition to the usual Gaussian beam optical tweezers, and greatly broadened the power of optical trapping technology in application to different situations. These various optical tweezers have been employed to manipulate and even drive dielectric particles, metal particles, and Janus particles in solution with high spatial resolution and accuracy by modulating the laser beam or shaping the geometrical and physical properties of particles. In particular, we show that Janus particles with different optical properties can exhibit the full potential of strong coupling in rotational and translational motions. The controllability and flexibility of Janus particles make them ideal candidates for general applications in nano- and microdevices, such as micropumps, microvalves, or micromotors that provide power to other devices. Finally, we discuss that in addition to measure the mechanical properties of biological macromolecules, the advancement of optical tweezers has been extended to investigate biological processes in vivo. Although the measurement and operation of these living organisms in vivo have faced great challenge and difficulty, optical tweezers can no doubt provide the opportunity for characterizing the dynamics of these life systems.

In the past thirty years, the science and technology of optical tweezers have made big progress, yet, with the input of knowledge from other research fields such as physics, optics, nanophotonics, nanoscience and nanotechnology, optical tweezers are still energetic and have continuously shown the power to break one by one physical limitations to this technology. These breakthroughs include manipulating nanometers dielectric particles and micrometers metal particles, capturing particles in both static and dynamical ways, and achieving complicate coupling motions. Moreover, when in combination with optical imaging and holographic technique, it becomes an excellent tool for directly probing various hydrodynamic and optomechanic properties of complex micro-objects in an aqueous environment. With the recent abundant advances, optical tweezers have generated a variety of applications, such as particles sorting, force transducer, morphology probe, and the embryo of micromachines. Meanwhile, novel beams have emerged by carefully constructing the properties of electromagnetic field and are introduced to enrich the means of optical tweezers. Furthermore, more and more functional materials and composite materials have been introduced to combine with the technology of optical trapping to explore fruitful mechanical interaction properties of these materials. Many novel optical phenomena have emerged and they had greatly help to reveal new frontiers of light–matter interactions and arouse vast application prospects. Such a technology is bound to pave the way for diverse promising applications in biosciences, physics, and material engineering fields.

Nevertheless, these studies are just the tip of iceberg. Note that futuristic applications of optical tweezers are mainly in biology field. There still exists a critical problem that in the microscopic world the Reynolds number of fluid is very small, much less than 1, and then the fluid will exhibit Stokes flow, namely laminar flow, where the viscous forces of fluid dominate inertial forces. However, laminar flow is a flow regime characterized by high momentum diffusion and low momentum convection. At the same time, taking the influence of external factors into account, such as Brownian motion of particles, thermal effect, and interference of other particles, it will be a hard task to comprehensively analyze the light–particle interaction in optical tweezers. In addition, as potential light-driven devices, trapped particles have the ability of energy conversion and mechanical output, and can achieve specific mechanical properties, such as directional transport, controllable rotation and so on. Therefore, it is an important scientific issue to investigate how to achieve high efficiency energy conversion (from electromagnetic energy to mechanical energy) and high mechanical output.

Furthermore, the improvement of spatial and temporal resolution with optical tweezers can greatly promote our capability to monitor various physical parameters, giving the precise space-time configuration and evolution of particles in focused optical field. Finally, the general, accurate, and efficient theoretical and numerical calculations [25,143,144] of optical forces and torques should be carried out, and this requires development of novel more powerful theoretical and numerical approaches. The calculated torque and force will be helpful for understanding the underlying physical mechanism of light–particle interactions, laying foundation for flourishing applications in controllable motions of micromachines.
